# Decoding the Influence of eHealth on Autonomy, Competence, and Relatedness in Older Adults: Qualitative Analysis of Self-Determination Through the Motivational Technology Model

**DOI:** 10.2196/56923

**Published:** 2024-10-30

**Authors:** Lynne M Cotter, Dhavan Shah, Kaitlyn Brown, Marie-Louise Mares, Gina Landucci, Sydney Saunders, Darcie C Johnston, Klaren Pe-Romashko, David Gustafson, Adam Maus, Kasey Thompson, David H Gustafson

**Affiliations:** 1 School of Journalism and Mass Communication University of Wisconsin - Madison Madison, WI United States; 2 Center for Health Enhancement Systems Studies University of Wisconsin - Madison Madison, WI United States; 3 Department of Communication Arts University of Wisconsin - Madison Madison, WI United States; 4 Department of Industrial and Systems Engineering University of Wisconsin - Madison Madison, WI United States

**Keywords:** self-determination theory, usability, mobile technology model, aging, eHealth, mobile health, mHealth, smart displays, video calls, older adult, chronic conditions, mobile phone

## Abstract

**Background:**

Older adults adopt and use eHealth systems to build autonomy, competence, and relatedness and engage in healthy behaviors. The motivational technology model posits that technology features, such as those on websites, smart displays, and mobile phones, must allow for navigability, interactivity, and customizability, which spur feelings of self-determination and intrinsic motivation. We studied ElderTree, an online system for older adults that provides on-demand videos of healthy living content, self-monitoring, and weekly researcher-hosted video meetings.

**Objective:**

We aimed to understand the theoretical crossover between the motivational technology model and self-determination theory using features of ElderTree to understand the usability of the technology and how it may support older adults’ autonomy, competence, and relatedness.

**Methods:**

Drawing participants from a randomized controlled trial of a mobile health app for older adults with multiple chronic conditions, we conducted qualitative interviews with 22 older adults about their use of the app; the interviews were coded using qualitative thematic analysis.

**Results:**

Older adults did find that features within ElderTree such as content available on demand, good navigation, and weekly researcher-led video calls supported feelings of autonomy, competence, and relatedness, respectively. Individual differences such as a background using computers also influenced participants’ experiences with the smart displays.

**Conclusions:**

Participants confirmed the features that increased internal motivation, such as interactivity correlating with feelings of relatedness, but they also found other ways to support autonomous health behavior change beyond narrow views of navigability, interactivity, and customization.

## Introduction

### Background

With the vast array of health resources available online, many systems to support individual-level health are available to the older adults who may need them. As older adults face a growing number of health concerns—chronic metabolic issues such as diabetes, hypertension, and obesity [1]; physical ailments such as pain or arthritis; and emotional concerns of loneliness and depression [2]—they increasingly seek such health information and services [3]. Web-based health technologies (eHealth) or mobile health (mHealth) technologies delivered through internet-connected devices focus on providing social support and increasing positive health behaviors [4]. One way to understand the adoption and continued use of eHealth technologies is using self-determination theory, which provides insights into the internal and external motivational factors that guide individuals’ drive to engage in healthy behaviors.

Self-determination theory, developed by Deci and Ryan [5], identifies 3 needs that drive motivation, specifically, the need for competence, autonomy, and relatedness. Competence is the ability for individuals to feel mastery over their environment, that the behaviors they want to do are within reach [6]. Increasing people’s perceived competence increases internalization of motivation to do a behavior and, thus, the likelihood of adopting it [7,8]. The need for autonomy describes the experience of volitional self-direction in thought and action. High levels of autonomy increase intrinsic motivation such that individuals feel more interest and enjoyment in the behaviors [7]. Autonomy is a key factor in many components of well-being as people with high levels of autonomy have lower rates of anxiety and depression and overall higher life satisfaction [9]. The relatedness factor is derived from the need to belong, highlighting how individual-level actions are significantly impacted by our need to be socially accepted and have regular contact with others and to have mutual care for another’s well-being [10]. Relatedness includes feelings that people of authority (eg, parents, teachers, and health care providers) provide respect and finding a sense of belonging within one’s peer community [6]. While all 3 components function independently, they also support each other in increasing intrinsic motivation [5,11].

In the context of health technologies, enhancing self-determination can increase an individual’s self-efficacy and commitment to healthy behaviors [4,12,13]. Broadly, the impact of self-determination theory on various measures of overall health has been well documented in several meta-analyses [9,14,15]. For example, self-determination factors increase positive health behaviors in the realms of exercise [16,17], mental health [18], and health communication [19]. However, understanding the specific components of eHealth and the way in which they influence autonomy, competence, and relatedness would greatly enhance practitioners’ ability to target features that increase positive health behaviors. The goal of this study was to examine how older adults engage with an eHealth tool, describe specific features of the tool, and consider the technological affordances of those features that can support older adults’ self-determination, as well as individual-level differences that may influence how the affordances impact the user experience.

### Motivational Technology Model and eHealth Applications

**Figure 1:**
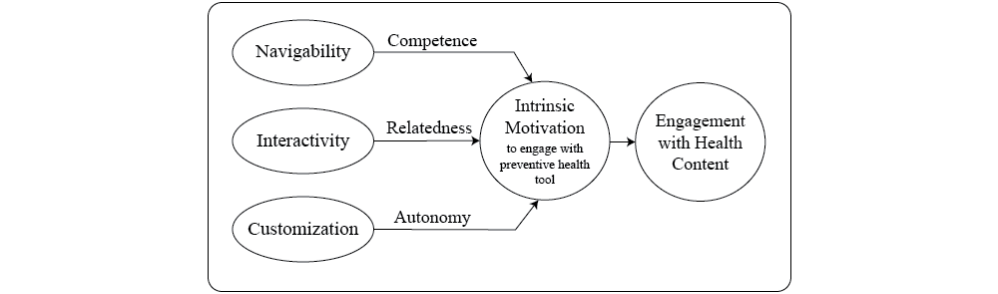
Theoretical motivational technology model of affordances that increase self-determination and promote preventive health behaviors, a focused view based on the work (reproduced from Sundar et al [21], which is published under Creative Commons Attribution 4.0 International License [32]).

The motivational technology model (MTM) is a framework for understanding how technological affordances or perceived and actual properties of the technology that shape and structure its possible uses [20] may impact self-determination. The MTM specifically supports ways in which technology features influence an individual’s self-determination (Figure 1 [21]) within the broader framework of the theory of interactive media effects (TIME) [22]. Sundar et al [21] identified technological affordances of navigability, interactivity, and customization that can improve an individual’s self-efficacy and intrinsic motivation to use the application for health improvement. Navigability describes how features allow users to easily search for and find the relevant content, which in turn can increase feelings of competence, decrease cognitive load, and improve user satisfaction [23,24]. Features such as menus, search functions, and breadcrumbs and being able to navigate to particular pieces of content all add to navigability [25]. Interactivity, in part, highlights the way in which technology connects people to increase their sense of relatedness. Such computer-mediated peer communication, both receiving messages from others and posting messages to others, shows promise in improving health and well-being [26-28]. Customization refers to how much an individual can make choices about the content or interface, allowing changes to what is available through user-driven tailoring, which increases a sense of autonomous motivation [21]. The MTM theorizes that eHealth tools support intrinsic motivation through the creation and use of technology that increases autonomy, competence, and relatedness, fulfilling needs that drive the motivation to pursue healthy behaviors.

Several studies in the literature specifically look at the overlap between the MTM and self-determination theory. These studies broadly support the links between technological affordances and self-determination [29], with evidence that interactivity increases feelings of relatedness but also competence and autonomy [25] and that customization could also increase all 3 components of self-determination [30]. For example, a cross-sectional study of smartwatches found that participants who rated the tools as interactive, navigable, and customizable also rated the tools as providing them with stronger feelings of relatedness, competence, and autonomy, respectively, and these increased engagement with the health apps [30]. A study of fitness apps using the MTM framework found that apps emphasizing interactivity increased feelings of relatedness, which ultimately increased motivation to engage in physical activity [25]. It also found that interactivity predicted all 3 components of self-determination and that relatedness mediated the relationship with health outcomes [25]. Another study found that the ability to customize physical health trackers increased all 3 components (competence, autonomy, and relatedness) in participants who used them, all of which increased engagement [30]. Finally, a qualitative exploration study using the MTM as a framework for designing a new mHealth app for managing rheumatoid arthritis found trade-offs between navigability and customization, observing that more choices made the interface more complicated [31].

### Customization as a Tool for Increasing Autonomy

Many more studies have looked at specific eHealth and mHealth features that influence self-determination. For example, customization, such as self-monitoring and providing individualized feedback, is most commonly used in eHealth systems to increase autonomy [33]. Additional research has identified several ways in which customization has influenced feelings of autonomy. An experimental study on mHealth apps found that users’ subjective ratings of perceived interactivity, or the degree to which users could customize the content, were positively correlated with autonomous motivation [34]. User-personalized conversations can also increase user satisfaction and general engagement [35]. An mHealth app for increasing physical activity that manipulated customization of the interface was expected to change autonomous motivation. While using the app did not directly influence participants’ feelings of autonomy, they did experience moderated mediation through the feeling of perceived control, moderated by a need for autonomy [36]. In mHealth gaming, user customization and content personalization can be persuasive for some types of people [33]. A review of mHealth studies found that participants often requested the ability to personalize their technology and that, if customization options were not available, they opted for other technologies that allowed for customization options [37]. Overall, customization appears to be tied to autonomy, defined by choices within and across apps.

### Interactivity to Increase Feelings of Relatedness

Many mHealth apps use interactivity to motivate and support users, create a sense of relatedness and bonding, and encourage continued use. The need for relatedness in health and health care leads humans to find and connect with health care providers as well as other people with similar health conditions [38,39] and is a key component of many online health interventions. In mHealth games, for example, cooperation and competition increase relatedness, such as having collective goals, opportunities to cheer others on or share stories, leaderboards, and individual or group competition [40]. These social features are seen as highly persuasive at increasing positive health behaviors in mHealth [33]. While gamification features are sometimes used in certain types of mHealth apps, health care–focused mHealth apps typically refer to interactivity as one-on-one or one-to-many computer-mediated conversations with health care providers or other system participants, often those struggling with similar chronic illnesses [41]. In the peer-to-peer context, the expression and reception of messages, both via personal messaging and through public posting on social platforms, is related to increases in bonding, social connectedness, and feelings of well-being [26]. In contrast, conversations with providers are often centered on health concerns more than supportive, relationship-building exchanges [42]. The role of eHealth applications in connecting individuals with peers, support networks, and health care providers may support a sense of relatedness in different ways.

### Navigability to Increase User Competence

Navigability can describe the ease with which users can move through an interface, and through the creation of a user-friendly site, users feel competent in accessing the content [22]. One measure of competence is the degree to which individuals can navigate and understand the app or web-based tool. The navigability of mHealth apps correlates with increased feelings of competence [25]. Applications with navigable user interfaces obtain more users who use the app for longer compared to apps with complex and cluttered interfaces [43,44]. Notably, navigable apps with better user experience have also been found to be more expensive to build. Overall, specific design features to increase navigability include a simple navigation scheme, clean esthetic style, personalized feedback and tailoring, and user customization [44]. These features can increase usability and enhance the user experience, which may bolster user competence within the mHealth app.

Another measure of competence is the degree to which technology provides users with educational content and increases their knowledge about health topics. mHealth apps can build user competence by providing access to helpful content, knowledgeable or experienced people, or a space to reflect on that content, allowing users to seek subjects and sources that build competence. Doing so has improved competence in a range of contexts: healthy eating [45,46], dementia care [47], and cancer care [45,48]. This same relationship was not observed for sexual health [49] or heart failure [50], suggesting limits to information subsidies.

### Intrinsic Motivation and eHealth for Older Adults

From this evidence on the ways in which eHealth affordances of customization, interactivity, and navigability can influence participants’ feelings of self-determination and their overall well-being, we expect that older adults, too, would experience increased self-determination when using eHealth tools; however, there are likely some differences from the general population. These differences might be due to age, engagement with eHealth broadly, and the ways in which self-determination is experienced differently by older adults.

Individual person-level characteristics may determine to what degree autonomy, competence, or relatedness create and sustain health behavior change. Advancing age is a significant predictor of reduced technology use [51], health behaviors, and overall health outcomes [52-54]. Older adults are much less likely to adopt eHealth and mHealth technologies [13] unless they have specific utility, such as offering needed social supports [55]. Generally, older adults are most likely to adopt mHealth when they perceive the technology as useful and feel confident in their ability to use it [37].

Higher perceived self-determination for engaging in health behaviors is correlated with improved health status among older adults [56] and linked to increased adoption of mHealth [57]. Age also has a significant impact on need for autonomy, with older people being more autonomy focused [58,59]. Finally, a study about Facebook use and older adults’ perceived self-determination and well-being found that the affordance of customization correlated with autonomy; interactivity correlated with relatedness; and, broadly, both correlated with enjoyment of using Facebook [29]. In addition, only feelings of competence correlated with increased well-being; however, Facebook is not an eHealth platform, so these increases were not expected.

### ElderTree: An eHealth Application

ElderTree, a website and eHealth application for older adults, provides on-demand videos of healthy living content, self-monitoring, and weekly researcher-hosted video meetings (ie, weekly meetups) [60,61]. The application effectively increases individual well-being and self-determination through encouraging social connections and providing health-focused content [62]. Users have a profile viewable to other study participants and can share content or chat with other users. ElderTree provides weekly updated health content, including blog posts (eg, about hearing aids, alcohol, and sleep); chair-based exercise videos for increased mobility, flexibility, and strength; guided meditation; and other content relevant to older adults, such as finance or just-for-fun videos [11,62]. A weekly health tracker helps users monitor their health in areas such as sleep, mood, medication adherence, and falls. Screenshots are available in Multimedia Appendix 1.

A key part of current ElderTree research is identifying how best to provide content to older adults, so participants are randomized to receive either a laptop or a Google Nest Hub Max smart screen. On the laptop, participants access ElderTree through a web browser. On the smart screen, they access an app triggered by the command “Hey Google, living well.” Both systems are touch screen based. Although ElderTree has been designed and studied as a tool for increasing self-determination, the specific technical features that can increase autonomy, relatedness, and competence and their role in self-determination have not been examined. Thus, we pose the following research questions (RQs):

1. In what ways do participants report that ElderTree, including an eHealth tool and weekly video calls, supported their autonomy, competence, and relatedness? (RQ1)
2. In what ways did the constructs of self-determination theory—autonomy, relatedness, and competence—map to the affordances of customization, interactivity, and navigability within ElderTree? (RQ 2)

## Methods

### Study Design

This study drew participants from the ElderTree study, a randomized controlled trial of adults aged >60 years with multiple chronic health conditions (N=216). Participants were randomized to use the evidence-based intervention (ElderTree) either on a smart display or on a laptop. The full research protocol is available in the work by Gustafson et al [61]. While the original study intended to enroll participants for 12 months, a change in the Google Nest Hub Max interface made the system incompatible with the ElderTree eHealth tool. The study was subsequently ended early and considered a pilot test.

### Recruitment

We conducted 22 interviews with participants from the original ElderTree sample pool. This number was selected to allow for enough variation and is generally considered an appropriate sample size for in-depth qualitative interviewing [63,64]. To select participants to interview, the research team first stratified all participants by research arm—laptop or smart display—and by weekly meetup attendance records—people who did not attend the weekly meetups, people who attended the weekly meetups but did not often speak, and people who attended the weekly meetups and most often spoke. In total, 4 to 5 participants from each group were contacted for interviews, capturing a wide variety of participant engagement and experience. Participants were contacted via email, SMS text message, and phone call. Interviews were conducted and recorded, with permission, by the lead author and held over Zoom (Zoom Video Communications) or phone call using a semistructured interview guide [64] after participants gave verbal consent to participate.

The interview guide was developed iteratively by all study authors to identify how participants experienced the affordances of navigability, customization, and interactivity. The interview guide first asked participants generally about their experience using ElderTree, motivations for joining the study and using the tool, how they felt about the navigational experiences, their experiences customizing the tool, and their use of the features that supported interactivity. Participants were also specifically asked about their experience with the weekly meetups. The complete interview guide with probes is available in Multimedia Appendix 2.

### Ethical Considerations

To ensure participant privacy and data security, participant contact information remained separate from all other information and on a secure server. Interviews included only participant ID numbers, and all identifiable information was removed from transcripts and notes. Participants were compensated for study enrollment by receiving either a laptop or a smart display, 12 months of internet service, and US $10 for each of up to 4 surveys throughout the study period. The study protocol and interviews received ethics approval from the University of Wisconsin Health Sciences and Minimal Risk Research Institutional Review Board (reference 2020-0868). Survey data were collected in REDCap (Research Electronic Data Capture; Vanderbilt University) as part of the original grant [65].

### Coding Process

To code the interviews, the authors used qualitative thematic analysis [66] through inductive and deductive coding to capture relevant themes [67]. The first author started the codebook with themes and codes from self-determination theory [6] and the MTM [21], adding more codes by reviewing the data and finding other salient themes. Coauthors then reviewed and modified the codes. To establish interrater agreement, 2 interviews were microcoded for specific features by 2 reviewers separately and then reviewed together to discuss and resolve disagreements [68]. A total of 2 authors then jointly reviewed 2 additional interviews and discussed the coding schemes identifying other potential new codes. When new codes were identified, all interviews were then recoded for those additional codes. Both reviewers coded all interviews and then combined and deduplicated their codes for final analysis. Data were analyzed using NVivo (version 1.7.1; Lumivero).

## Results

### Participants

The overall response rate was 88% (22/25) of the contacted participants. Interviews lasted an average of 33.1 (SD 10.4; range 10-50) minutes. Participants, who were aged between 61 and 92 years, were predominantly female (14/22, 64%) and White (17/22, 77%; Table 1).

**Table 1:** Sociodemographics and participant characteristics by device type (N=22).

			Laptop (n=13)		Smart display (n=9)		Overall
Age (y), mean (SD; range)			69.4 (7.3; 61-84)		70.1 (9.6; 62-92)		69.7 (8.1; 61-92)
**Sex, n (%)**							
	Female	9 (69)		6 (67)		14 (64)	
	Male	4 (31)		3 (33)		7 (32)	
**Race, n (%)**							
	African American	3 (23)		0 (0)		3 (14)	
	Asian	0 (0)		1 (11)		1 (5)	
	White	10 (77)		8 (89)		17 (77)	
**Educational level, n (%)**							
	High school	2 (15)		1 (11)		3 (14)	
	Technical school	0 (0)		1 (11)		1 (5)	
	Some college	3 (23)		2 (22)		5 (23)	
	College graduate	5 (38)		4 (44)		8 (36)	
	Postgraduate or professional	3 (23)		1 (11)		4 (18)	
**Social ties, n (%)**							
	Do you have a significant other?	4 (31)		5 (56)		9 (41)	
	Does anyone else live with you?	7 (54)		5 (56)		11 (50)	
**Engagement with weekly meetups, n (%)**							
	No or limited attendance	4 (31)		4 (44)		8 (36)	
	High attendance, low participation	3 (23)		2 (22)		5 (23)	
	High attendance, high participation	6 (46)		3 (33)		9 (41)	
**Comfort with technology, n (%)**							
	Has a smartphone	11 (85)		7 (78)		18 (82)	
**Has ever used a smart display, n (%)**							
	Never or rarely	5 (38)		6 (67)		11 (50)	
	Sometimes, often, or very often	8 (62)		3 (33)		11 (50)	

### Linking Self-Determination Theory and the MTM

#### Customization and Autonomy

Overall, participants discussed several features that led to feelings of autonomy, including content availability and system customization (Table 2). Participants reported customizing when they wanted to exercise and watch curated health or exercise videos and how the content being available anytime supported their autonomous use of the system, particularly for the participants in the laptop condition:

Oh, two or three o’clock in the morning I’ll be on, on the, you know, the computer, and it was just so good...It’s just there and it’s available. I love that.

P06; laptop

Participants also liked that they were able to access the content themselves without requiring talking to others, again supporting their autonomy:

I think it has [helped me] because...it’s something to go to without bothering anybody or needing anybody’s help.

P25; laptop

Participants also customized how they used the application and what they used it for. The ability to use it for many different purposes was present both in the laptop and the smart display group, with participants using it to generally browse the web—“The fact that it, you know, I was able to get to all websites and not just ElderTree, that’s another bonus. I’m all over the Y[MCA] and everything” (P06; laptop)—or during the holidays to play Christmas music through the smart screen*.* The ability to select parts of the intervention that suited their needs was important, allowing them to set goals and engage in physical exercise either with or without the app:

Well, I watched them to get the general idea of how to do [the exercises] on my own, if I can do it on my own, and I wouldn’t have to actually watch it...[I integrated] the exercises in my daily routine or whatever. Like cooking, or if I’m walking in the house, I’ll be incorporating the instructions into what I’m doing, like balancing.

P23; laptop

Participants used ElderTree to relax and were happy to have a tool that could help them do so:

I would go back to ElderTree after gardening in the evening, to relax.

P06; laptop

It was nice to do the calming exercise. It just took ten minutes to settle my brain...When I wanted to relax, I would listen to the ones that had you close your eyes...the next thing I knew I was asleep!

P15; laptop

Participants were able to control their mood by customizing what type of content they accessed on the devices.

**Table 2:** Components of ElderTree that seemed to support user autonomy, competence, and relatedness and the number of participants reporting that experience by device type [69] (N=22).

Self Determination Theory construct and applicable device types^a^				Description		Participants, n (%)
**Autonomy**						
	**Use customization**					
		*Laptop*	Accessing content whenever (eg, in the middle of the night) and wherever (eg, bringing the laptop to another state to use it or moving from room to room)		13 (59)	
	**Content customization**					
		*Laptop and smart display*	Using the system as desired, including outside ElderTree (eg, music, images, and searches)		2 (9)	
**Competence**						
	**Content access and navigation**					
		*Laptop and smart display*	Being provided with quality content without searching		9 (41)	
	**Content navigation**					
		*Smart display*	Figuring out the system and how to navigate		13 (59)	
	**Health competence**					
		*Laptop and smart display*	Having access to regularly updated exercise, meditation, and entertainment content		9 (41)	
		*Laptop and smart display*	Using the system to learn about physical and mental health, aging well, and managing pain		3 (14)	
	**Technology competence**					
		*Laptop*	Bringing previous experience with computers and technology to navigating and using the system		8 (36)	
		*Smart display*	Finding excitement and enjoyment in learning new technology		4 (18)	
**Relatedness**						
	**Weekly meetups**					
		*Laptop and smart display*	Connecting with other participants		15 (68)	
		*Laptop and smart display*	Seeing other participants		4 (18)	
		*Laptop and smart display*	Chatting in small breakout groups		2 (9)	
	**Weekly tracker**					
		*Laptop and smart display*	Talking to health care providers about ElderTree and the weekly self-report health status		6 (27)	
	**Asking others for help**					
		*Laptop and smart display*	Connecting participants with others who could help them, including connecting with study staff		3 (14)	
	**Discussion boards**					
		*Laptop and smart display*	Reading what others wrote and finding new information		3 (14)	

^a^Device type has been italicized.

#### Features That Detracted From User Autonomy

Table 3 reports specific counts of features that participants reported as detracting from feelings of autonomy, competence, and relatedness. Participants described feeling less autonomous when they were unable to customize the tool to their liking. The specific customization feature of favoriting content for later was used by only 5% (1/22) of the participants. Many did not know about the “favoriting” feature when asked whether they used it, and the participants who did know about it found it frustrating:

Instead of having to do down the tree to go find it, you could add [a video to] favorites and you could have “favorite” ones that you do all the time...but that didn’t work either.

P09; smart display

There were other customization options that participants might have liked. One participant, for example, discussed wanting more self-tracking options, such as for blood pressure. Participants also wanted the smart display to be portable, saying that “It felt a bit outdated...[the smart display] had to be plugged in, and it was not mobile. We are so used to everything being mobile now” (P04; smart display). The difficulty in customizing the location felt constricting for participants.

**Table 3:** Components of ElderTree that seemed to detract from user autonomy, competence, and relatedness and the number of participants reporting that experience by device type (N=22).

Self-determination theory construct and applicable device types^a^			Description	Participants, n (%)
**Autonomy**				
	**Interest**			
		*Laptop and smart display*	Not being interested in using ElderTree	4 (18)
	**Content customization**			
		*Laptop and smart display*	Finding the tool and content not customizable to their needs or wanting more customization options, such as tracking blood pressure	4 (18)
**Competence**				
	**Technological issues**			
		*Laptop and smart display*	Experiencing technical issues using the system, accessing content, or joining the weekly meetups	14 (64)
	**Previous work experience with computers**			
		*Laptop and smart display*	Not having previous experience with computers or technology	7 (32)
	**Content newness**			
		*Laptop and smart display*	Finding the content stale or not growing with participants’ abilities	6 (27)
	**System navigation**			
		*Smart display*	Finding the tool difficult to navigate	3 (14)
**Relatedness**				
	**Weekly meetups**			
		*Laptop and smart display*	Feeling too old, too young, or too healthy for the group by comparison	3 (14)
		*Laptop and smart display*	Feeling bored or otherwise just not interested in connecting with other participants	7 (32)
		*Laptop and smart display*	Feeling like the meetings were too large	4 (18)
	**Weekly tracker**			
		*Laptop and smart display*	Being unable to connect with health care providers about their weekly tracker results (ie, finding that providers did not care)	7 (32)
	**Discussion boards**			
		*Smart display*	Not having knowledge about the feature	4 (18)

^a^Device type has been italicized.

#### Navigability and Competence

While there were a few examples of how application navigability was related to a participant’s feeling of competence using it, that was not the only way in which they talked about feeling competent with the technology. Participants generally did not discuss the navigability of the application much, but when prompted, many said that they had no problems finding what they needed:

Yeah, I did, the ElderTree site was pretty straightforward.

P24; laptop

...it took a little bit to figure it out but eventually I figured it out.

P31; smart display

...if [the technology] is complicated, I’m not prepared to learn.

P04; smart display

In this way, competence and navigability were connected. One participant described feeling competent because they did not have to search through a huge number of videos to find ones applicable to them, in contrast to a public website such as YouTube, where they felt less able to find the quality of videos that they were looking for:

I was interested in specific kinds of topics that I was able to find. I mean if you go to YouTube, you can find videos about how to maintain your balance, or work at your balance, but it’s so disorganized. Because that’s curated, and I guess that’s a real benefit of what something like ElderTree accomplished.

P25; laptop

Familiar user interfaces and navigation cues on the laptop created feelings of competence, such as using the touch screen:

I’m glad I was in the laptop group and was able to do more on a computer. I used the touch screen functionality, I preferred it, but I could use the mouse.

P53; laptop

Another participant reflected how it was easier to correct errors using the touch screen, which made the experience more enjoyable:

They gave me the mouse and the pad but it’s all touch screen...I can just touch it and then like backspace or whatever I need to do and then fix it and go on...It’s so awesome for me because that’s what I was looking for and, I never, you know, knew that I would enjoy that as much as I do, it just makes it so easy for me.

P25; laptop

While some of the smart screen participants had initial trepidation, many had positive experiences after becoming familiar with it, such as one participant who stated the following:

It took a little bit to figure it all out but eventually, I got the swing of it. [The smart screen] was kind of fun, something new to figure out.

P31; smart display

Other participants described how fun it was to both figure out the new technology and find the content:

It was kind of a novelty to have this little piece of equipment that would have a little message every morning, or day depending on what time you listen to, looked at it...I liked going in there and...doing some of the exercises,also, um, asking it to play certain music.

P12; smart display

Participants sought new content along with new technologies, and it was the system navigability that supported their desire to try out all the existing content or a drive to look at the application regularly to see when new content was introduced:

Overall, I tried every single [exercise video], and some of them I did twice or three times...I tried out everything.

P01; laptop

This supported their competence with finding and using health information:

I wanted to see what everything was. Late nights, at night, I would do some of the meditations, and movement, too. I liked exploring and was glad to do that because I found things I otherwise wouldn’t see.

P53; laptop

Several participants mentioned that regularly updated content was a motivator for checking and using the app, such as the inspirational quotes or new content:

You know, and I tried, whenever they said something new was on it, I tried to go to it sometime during the week.

P29; laptop

The new content motivated them to initially log on to the system and then often kept their attention.

Finally, the content on ElderTree supported users’ competence and knowledge of specific health topics, particularly the modules on pain:

A lot of us deal with pain. They had good information about pain, and we would use that information.

P01; laptop

Another participant found that, again, the pain modules provided important information for how they could handle their pain:

There was some videos that went into a lot more detail about pain [on ElderTree] and...it was really interesting.

P15; laptop

Inherent in the ability to learn about how to manage pain was the ability to find and watch the video segments about pain or other topics of interest. Participants also learned how to do physical activity movements that could help manage and reduce their pain:

Every night, I’d sit there and do two or three of those [mindful movements videos on ElderTree]...to get that shoulder moving again.

P09; smart display

#### Features That Detracted From Competence

Within the system, there were components that the participants discussed negatively, particularly in the smart screen condition. The participants with a smart screen mentioned various issues, such as not being able to log in or their camera not functioning properly for the meetings, which hindered their interest in using the application by reducing their feelings of competence. They described how they wanted to be a part of the video calls or do the exercise videos but that they were challenged by the system and some technological issues:

To have to go through all the, all the hoops to get the ah, Google display thing to work...I do have computer skills. It’s like, I actually used the original internet back in the seventies.

P48; smart display

Another participant found the content helpful but the system cumbersome, so they went outside of it:

A lot of the videos I found on YouTube...I wouldn’t even try to mess with [ElderTree]. The only time I would use it with ElderTree was when we were online [for the weekly meetups].

P48; smart display

Technical troubles were likely to deter participants.

Stale content was also a deterrent for some participants. For one, because new content prompted participants to look at ElderTree and browse, participants mentioned forgetting to use the application when they did not expect anything new, with one participant saying the following:

I can’t be bothered, because it’s not new, it’s not on your mind, you don’t think about, don’t bother going back and having a look at it.

P04; smart display

Other participants felt like the content needed to progress with them, to become more challenging as they became more able, such as the following participant, who used ElderTree a lot while they were healing but not as much after they recovered from their surgery:

After my shoulder got better, I needed more. I tried the balance ones, but the content got stale.

P09; smart display

New content engaged participants, but not having content that grew with them deterred participants from regular use.

#### Interactivity and Relatedness

To examine the role of ElderTree and the device regarding relatedness, the interviewer asked questions about the weekly meetups and the online discussion board. Table 2 reports resulting counts of specific features that participants reported as supporting autonomy, competence, and relatedness. The researchers found that technological features that increased the interactivity affordance, and participants’ feelings of relatedness were closely linked concepts. In times when technology afforded interactivity through an interactive social experience, participants were able to connect with others, interested in doing so, and discussed the importance of that connection. While participants never met in person, nor were they required to attend the weekly meetings, they often found the meetings to be a very impactful part of the study. The meetings connected participants with each other and were discussed as an experience that supported their feelings of being closer to the people in the group. They discussed that being in the meetings gave them “the knowledge that you’re not alone and that there are other people fighting through difficulties. It’s a whole lot different knowing that than sitting at home by yourself thinking you’re the only one going through it” (P29; laptop). Regardless of their similarities or differences, participants felt like they were a part of a group, saying that “Whatever condition you are in, it gives you a way to socialize” (P04; smart display). The participants identified that the group was diverse:

It was uplifting to talk to different people...I liked the conversations. Over the phone we got pretty close. We never met, but we had a good group, for such a large group.

P40; laptop

The weekly meetups provided opportunities for older adults to learn from others:

[The ElderTree Meetup] helps you mentally because just hearing about what other people are going through, and what things have helped them, what resources they used, was very helpful.

P07; smart display

Another participant commented that “ElderTree makes us thankful...it gives you another perspective on things” (P22; laptop). The interactivity experienced through the weekly video calls seemed to correspond with feelings of relatedness by providing users with a social network to corroborate the difficulties of growing older and resources for new and ongoing health and social issues.

There were certain technical features as well that increased relatedness by giving participants specific ways to interact with each other, such as the hand-raising function that created organization regarding who could speak:

I’m a little shy about speaking up and it can be hard for me but um, it was nice that they had the hand raising function [on Google Meet], because that helped a little bit to be called. So, sometimes, I would wait and then if it seemed like there weren’t a lot of people talking, I would raise my hand.

P15; laptop

The chat feature within the breakout groups also garnered positive responses from participants, who said that “We had a good time with the chat feature” (P01; laptop) but that they wanted more—“It would be nice to have a chat option, a one-on-one chat within the meetings” (P15; laptop)—to support a smaller community. The breakout groups also created a closer sense of community by encouraging a smaller group to come together and chat:

I like the fact that we could all contribute first thing, you know, I mean when they get the breakout groups that was nice because I need a chance to offer what was good for the week and, you know, one good thing or one, you know, thing that wasn’t so good.

P5; smart display

These structural components supporting interactivity seemed important to participants’ feelings of relatedness to other participants and their general engagement with ElderTree.

A few unexpected features of the study design supported participants’ feelings of relatedness. Participants reported that emailing or calling study staff when they had a question was particularly helpful, as one participant said:

[The ElderTree study staff] were really great. We’d go to them with any question...there was always somebody there that would help.

P30; smart display

In addition, participants talked about the importance of the racial and age diversity on the calls, as one participant reported:

I am a white person, and I have some interaction with people of other races and backgrounds but not necessarily that much. It was good for me to listen to the gentleman and the ladies that were of other races.

P07; smart display

#### Features That Decreased Relatedness

While many participants appreciated the diversity, some reported feeling like outsiders if they were not like other participants because they were older or healthier than most participants. Specific features of the system were also brought up when asked about whether they felt that they related to other participants. For example, participants reported being uncomfortable not being able to see the other participants when their videos were not on or when there were technical difficulties so they could not share their own video streams:

I could get in and my camera wasn’t working, so, and I don’t like, I like being able to see it if I’m going to be talking to people, I like to have them to be able to see me as well.

P30; smart display

There were people who didn’t even have their cameras on. I could only see a handful with video on, many with videos off the whole time. I always had the video on.

P24; laptop

The anonymity affordance [20], in which participants could turn off their camera during the video calls, may have decreased a sense of relatedness for some participants who would engage only partially:

At the end of every meeting, you know, we did...an exercise dance or whatever, but you do it sitting down. I do not know if it’s my own self-consciousness or whatever, but usually I would do it up to a point, but I would turn the video off.

P24; laptop

There were a variety of reasons why participants did not want to have their cameras on, such as technical difficulties or because they did not want to share information about themselves, such as them doing an exercise video or showing the inside of their house:

I didn’t have my camera on because I was at my messy desk downstairs.

P26; laptop

One participant commented that it was difficult to truly connect with the other participants because they were only connecting online and not in person:

It’s hard to look at a video, to be engaged that way, you know, at a deeper level. It’s not as if we’re in the same room at a party or a live event where you get to see [people] It’s harder to replicate and it’s harder for me to open up...Maybe it’s just my generation, I don’t know.

P22; laptop

Finally, the weekly health tracker prevented some feelings of relatedness for participants who wanted to discuss it with their physicians:

I usually did [the weekly survey]. But I thought they were kind of ridiculous. Because, they were nice for me, but the few times that I got them and took them to my doctors, they didn’t give a ding-dang.

P29; laptop

Expecting their care team to want the data and finding out that they did not isolated the participants.

### Personal Factors That Influenced Intrinsic Motivation to Use ElderTree

Individual participant characteristics influenced how they used the system, such as having work experience with computers or having a learning disposition. Some participants came to the study with an established sense of technological competence gained from years of working with computers. Although many participants were already retired, several talked about how their job required them to use computers and smartphones. Competence in using the ElderTree platform for these older adults was, for some participants, unrelated to how it was laid out and, instead, a function of a participant’s lifetime of experiences, such as for one participant, who worked in technical services and would use video calls to troubleshoot issues. Other participants did not enter the study with a lifetime of work experience that involved interactive technology or digital media, and they lacked competence as they entered the study, such as one participant who stated the following:

My life was as a secretary, and I said no office machine was going to get the better of me, but the electronic age has.

P10; smart display

Other participants mentioned jobs as bus drivers and childcare providers or other jobs that were not completed on a computer, and for some of these participants, the system was not usable based on their past life experiences.

Another driver to use the system was participants’ desire to learn technology and engage with something new. Some participants found that the novelty of the technology was what prompted them to use it even if they had no particular health goal, such as one participant who said that his motivation for enrolling in the study was the newness of the technology:

I’ve wanted to learn to do more typing, learn a new technology.

P11; smart display

Other participants also positively portrayed their interest in the novelty of a laptop or smart screen or just learning how to incorporate new technology into their lives, such as the following excited claim:

I love [technology]. I’m all in. I try to learn as I go, utilize it and that’s where you keep your calendar and all appointments.

P06; laptop

Individual differences and preferences for exploring new technologies were often discussed by participants as reasons for engaging with or stopping use of ElderTree.

## Discussion

### Principal Findings

In attempting to understand the crossover between self-determination and the eHealth affordances of customization, interactivity, and navigability, we interviewed 22 participants who used either a smart screen or laptop for a 6- to 12-month period and asked them about their experience, things that motivated them to use the application, components that were useful, and whether and how they connected with other ElderTree participants. We used the frameworks provided by the MTM and self-determination theory to guide our collections and coding. Structuring our questions around core needs of autonomy, relatedness, and competence and tracing connections with customizability, interactivity, and navigability provided new insights into how these affordances shape how older adults use eHealth tools. The participants discussed how the ElderTree application specifically supported all 3 human needs that drive internal motivation. While having a platform that afforded interactivity through discussion boards and a hosted weekly meeting supported participants’ feelings of relatedness and got them moving with an exercise video, they also talked about how on-demand, curated videos and hardware that could move around their house allowed them to customize their use of the platform and gave them more autonomy over when they wanted to exercise. Some specific content on ElderTree that participants particularly found beneficial were the pain modules and the calming movements that encouraged stretching and breathing. However, many of the experiences that users had could be benefits of any health application that connects older adults with content and other participants. These benefits need not be unique to the ElderTree platform, but they do require more than just access to content. Confirming previous findings, directly engaging with other study participants and study staff was critical to supporting participant relatedness and general interest in the application [37].

Individual characteristics, such as technological competence, differing interest in the ways in which they could track their own health, and preferences in meetup groups, seemed to reflect differences in how participants used ElderTree. Older adults are closing the digital divide [51], and many are willing to try new eHealth and mHealth options [70]. However, there are individual differences in interest in experimenting with technologies, so considering not only the technological affordances of eHealth platforms but also the personal characteristics, such as through a measure of technological innovativeness [71] or technology adoption [72], may capture individual differences in older adults’ interest in the challenges of using eHealth [22] or interest in health-tracking applications [73]. Measures of technology self-competence [71,74,75] may also help describe some of the differences observed among older adults using eHealth to account for the technological comfort and competence that older adults bring to a study, such as being worried that they will break the device or make an irreversible change, and those fears have can have a significant impact on how they see the eHealth and potential outcomes regardless of how navigable it is. This means that some older adults will use the technology even if they do not feel comfortable with the navigation, whereas for others, that will be a barrier [37]. There are many ways in which technological features may impact older adults’ experience of using an eHealth application.

### Implications for eHealth Designers and Researchers

Future designers creating these technologies can consider that certain eHealth affordances support older adults’ intrinsic motivation to engage with health content, such as exercise videos, meditations, and social interactions. Interactivity is a useful way to support relatedness, and ElderTree used scheduled socializing and exercise time through the weekly meetups. While these were generally liked, future implementations could better engage participants through smaller groups that are organized around common interests or health concerns, a finding shared with those of other eHealth studies [31]. Customization, too, supports participants, and in this study, participants asked for several additional customization options, such as including more exercise content of varied difficulty and tracking additional health elements in the weekly tracker, and users in the smart device group asked for the ability to customize where they used the devices, a commonly cited request for supporting autonomy [76]. While we only explored the affordances of interactivity, navigability, and customization based on the MTM, there were several other concepts that may be considered in future work exploring affordances of eHealth applications. ElderTree afforded participants access to newly updated content, which they found was a motivator for engaging in positive health activities, such as meditation and physical activity, and in general can support older adults’ healthy aging [77]. Given the complicated nature of defining affordances [78], we defer the work of declaring the aforementioned features as such to future researchers.

The hardware also offered customization options, and future designers could consider technological affordances beyond just what is in the eHealth application itself. In this study, participants used the smart display to play music; read inspirational quotes; and view inspirational, beautiful pictures for enjoyment. People in the laptop group were able to use it for visiting other health- and non–health-related sites or to connect with their family and friends. These extracurricular uses should be considered for their potential to support participants’ engagement with health technology and opportunity to engender further health activities.

The last design consideration is that technology use, particularly by older adults, is done in a complex environment in which the other users and available support staff play a critical role in maintaining an individual’s interest in the eHealth application. In this way, designers may not be able to successfully deploy an eHealth application into an app store without staff and without synchronous ways for users to connect. ElderTree does this by providing users with unlimited support staff available via phone or email and through hosting weekly calls. Participants brought histories and experiences with technology and social connection and had different abilities and interests to learn and engage.

### Limitations and Future Research

This study has several limitations. First, the qualitative research provided a richer understanding of how participants experienced the application, but there was a wide variety within the population. We captured some of that variety in this study, but older adults are incredibly diverse [79], and we likely were not able to fully elucidate all the experiences that the participants had with the affordances of ElderTree. Furthermore, while we attempted to increase the racial and ethnic diversity of the sample, we were not able to reach as many participants of racial or ethnic minority groups as we would have liked. These findings may not reflect the experiences of minority populations, who are often left out of research on eHealth [80], leading to worsening digital divides. Ongoing ElderTree research is prioritizing enrolling a diverse population, and future qualitative studies will be able to reflect that diversity. Additional research should also consider the degree to which participants desire a homogeneous or diverse group, and the potentially different social groups and functions that older adults desire.

Another limitation is that participants likely experienced recall bias in what they remembered about using the system and what modules they used. ElderTree and other eHealth tools often show significant participant attrition [81] such that people use the system more when they first receive it but then stop using it. Future studies should use system logs to quantitatively measure participant use and the relationship between system use and participant self-reported autonomy, competence, relatedness, and well-being to help support the results presented in this paper. Finally, while this study used a Google platform for ElderTree, that platform is no longer compatible with ElderTree, and future studies will be conducted on alternative smart display platforms.

### Conclusions

The aging population continues to adopt new technologies, and we need insights to shape the design of eHealth applications to best support older adults. This study found that there was excitement among the participants to use new technologies such as smart screens, although some participants needed extra support to use them most effectively. Self-determination theory can help app designers build products that support older adults with both low and high digital literacy. For example, providing multipurpose technology, such as a laptop, can better support older adults who bring competence and autonomous interest in using it. The MTM and self-determination theory components overlap, but there are also personal differences that have strong influences on how older adults use technology. In this way, future research can include additional eHealth affordances and personal-level characteristics to support individuals’ self-determination in engaging in healthy behaviors.

## Appendix

Multimedia Appendix 1 Screenshots of the ElderTree system taken from the laptop condition.

Multimedia Appendix 2 Semistructured interview protocol used with ElderTree participants.

## Abbreviations

mHealth: mobile health
MTM: motivational technology model
REDCap: Research Electronic Data Capture
RQ: research question
TIME: theory of interactive media effects
