# Supplementary material for: Decoding the Influence of eHealth on Autonomy, Competence, and Relatedness in Older Adults: Qualitative Analysis of Self-Determination Through the Motivational Technology Model
Source: JMIR Aging. 2024 Oct 30;7:e56923. doi: 10.2196/56923 (PMC11561439; doi:10.2196/56923)

**Multimedia Appendix 1: Images of ElderTree**

Home screen of ElderTree website, accessed through the laptop browser.


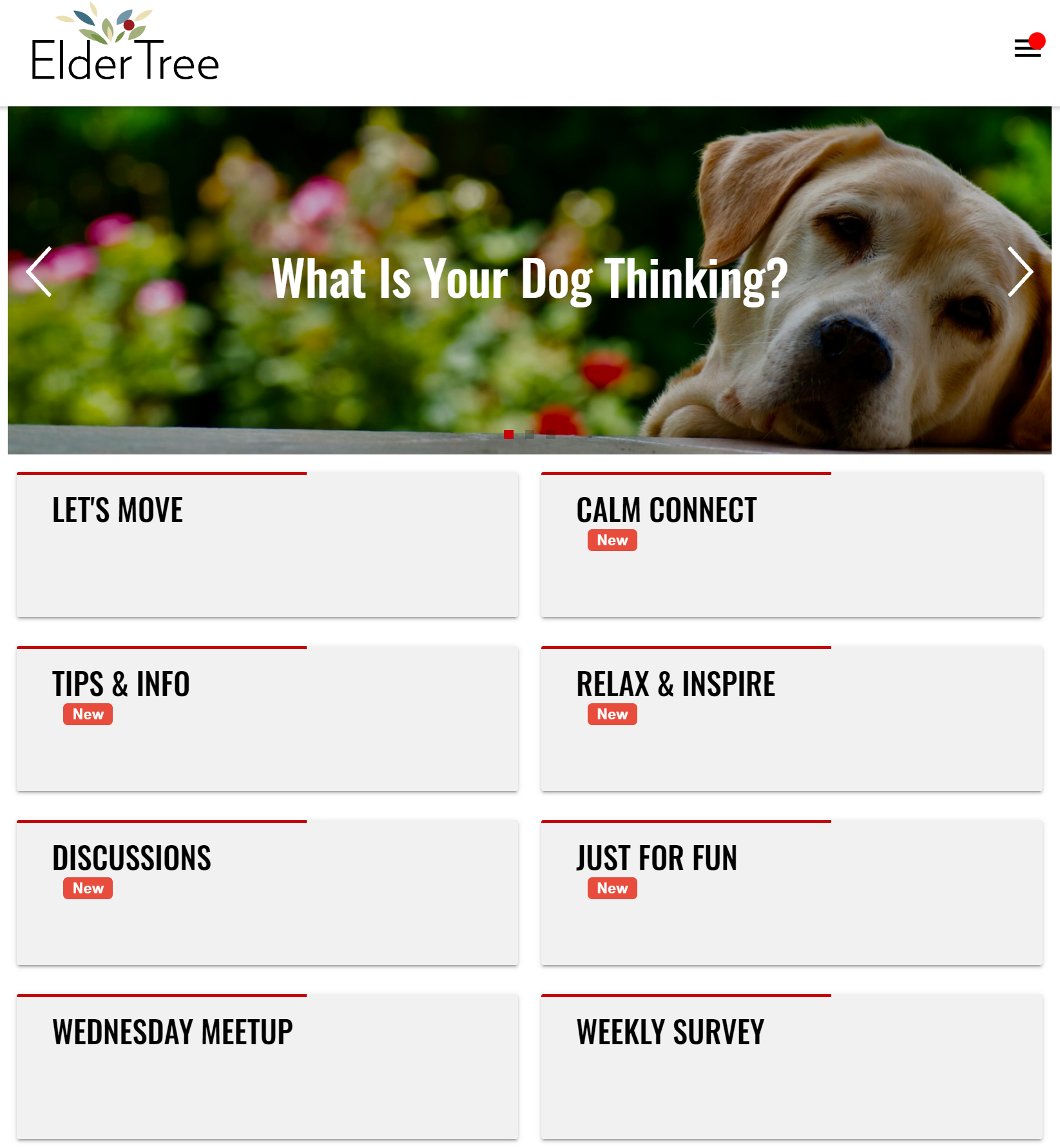


Screenshot of ElderTree system exercise videos, found under the “Let’s Move” section. Accessed from the Google Nest Hub Max.


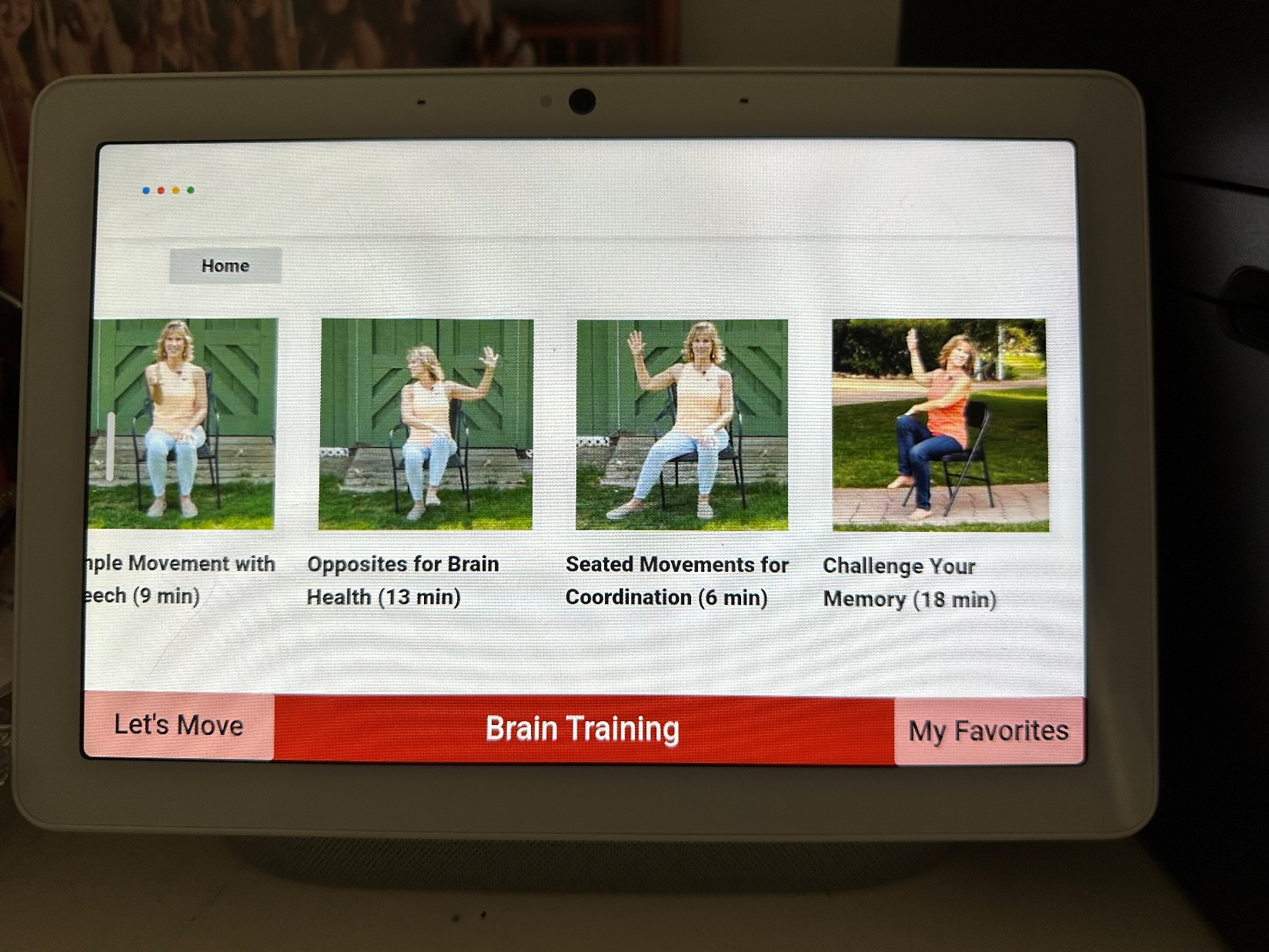

Supplement: Multimedia Appendix 1 [file aging_v7i1e56923_app1.docx]
