# Supplementary material for: Decoding the Influence of eHealth on Autonomy, Competence, and Relatedness in Older Adults: Qualitative Analysis of Self-Determination Through the Motivational Technology Model
Source: JMIR Aging. 2024 Oct 30;7:e56923. doi: 10.2196/56923 (PMC11561439; doi:10.2196/56923)

Technology Affordances and Socialization for Increasing Self-Determination

*Thank you for giving us some of your time today. We’re interested in hearing more about your experiences with ElderTree, particularly as a person who [was actively engaged in the weekly meet ups/had very high attendance at the meet ups/didn’t seem to attend the meet ups very often] We will be having conversations like this with 15-20 of the other participants. We are interested in hearing both about things you liked, and things you didn’t like. There is no wrong experience to have, and no wrong answers to give. I’m going to start with some questions about the [website/app], and then we’ll talk in depth about the weekly Meet calls. Just so you know, we are recording this so that I can go back and review our conversation. Is that OK? I’m also going to try to take notes as we talk so you may hear me pause or type as you’re responding.*

*Motivation*

1. To start, why did you join the study? Did you have any particular hopes for what you’d get out of ElderTree?

*Technology use*

1. *[For those in smart display arm]* You can use the app by either talking to it, or by touching the touch screen. How did you tend to use it?
2. How often did you use Elder Tree? Again, it’s totally fine if you never used it or hardly ever used it – we just find that really helpful to know. What’s your sense of how often you used it?

*If they said they used it regularly*

How did it fit into your schedule? Like, would you check it once a day, or once a week when you got the survey? Particular times of day?

*If they say they didn’t use it much,* That’s totally fine – that’s what we want to find out. So, how come you didn’t get to use it much? *Often they’re pretty busy and socially engaged and ET isn’t adding much value.*

Follow up – where did you have it in your house? How easy was it for you to get to?

1. I’m going to go through a bunch of things on ET and ask if you used them or not and whether you ran into difficult or weren’t interested or found them useful… Again, we’d love to know what was the case for you.
   1. Doing the weekly survey – did you tend to do those? Did you have any troubles?
      - 1. If yes – so what happened? Did it get fixed?
      1. Did you find the surveys helpful or frustrating in any ways?
      2. Do you think any of the things you tracked over time changed during the course of the study?
      3. After your weekly survey, you could see the graph showing the pattern over time. Did you ever see that?
   2. One option was to get a print-out of your survey results to take to your doctor’s appointments.
      1. Did you ever get those?
      2. Did you ever discuss it with any of your healthcare providers? Anyone else? What happened? How did it go?
      3. Did ET in general ever come up in conversations with your healthcare team?
   3. What about doing the modules about pain – did you get a chance to listen to any of those?
      1. Any problems or challenges using them?
      2. Any that you found helpful? Do you think you learned anything or not really?
   4. What about the rest of the tips & info sections…
   5. How about the Let’s Move section. Did you watch videos from there?
   6. What about the discussion group? Did you ever go there?...
      1. Did you ever post or were you mostly just reading?
      2. Did you find it interesting? Anything useful? Did it make you use other parts of the app?
   7. Did you get any notifications or messages just for you? Did they affect how you used the app?
   8. Overall, do you feel like you understood all the parts of ET and how to use them? Do you think you used it effectively?

*Social Interaction and Support*

1. Did you try the weekly meet-ups? If yes
   1. How often did you go?
   2. What were you hoping to get out of those?
   3. How did those feel for you? Were there things you liked or didn’t like?
   4. What was the group like? Did you feel like a part of it?
   5. How easy or hard was it for you to hear people? To see people?
   6. Do you feel like it had any effects after the meet-up? Did it encourage you to use any other parts of Elder Tree or do anything different?
2. Do you have any plans to continue meeting even without the ElderTree staff? Have you joined the Wednesday call?

**Overall sense of the project**

- 1. Do you think ElderTree had any effect on your pain or your health in general?
  2. Do you think it had any effect on your mood and the way you were feeling?
  3. What would make it better? Do you have any other suggestions to improve it for other people like you.

So, last thing, if you have time: just to get a bit of background

**How much did you use smart displays, phones before?**

**Do you use Facebook at all?**

**What about texting with family or friends?**

**Face Time or other types of video calls?**

Interview time: max 2 hrs.

Relevant Screenshots:


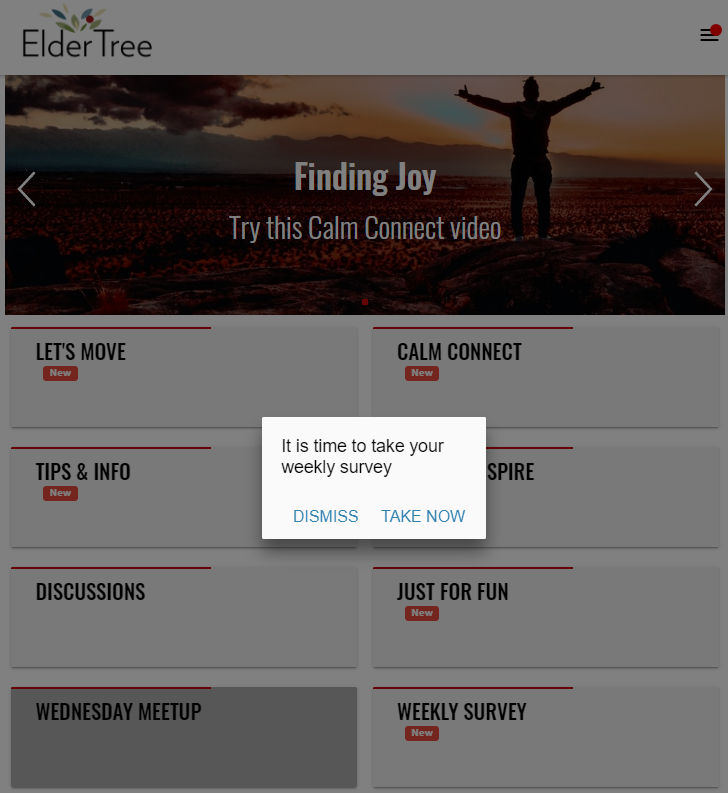


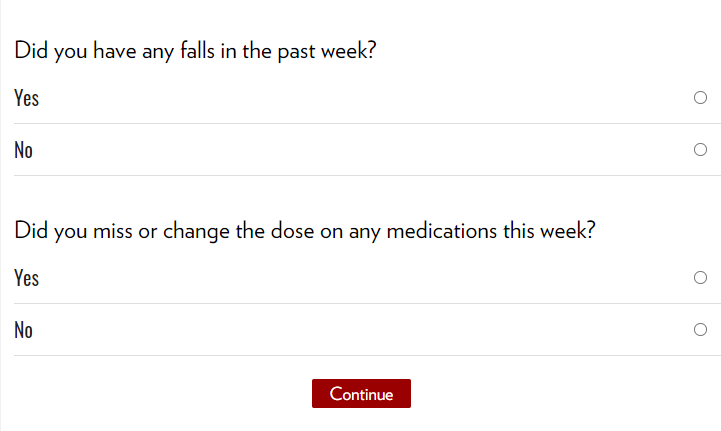


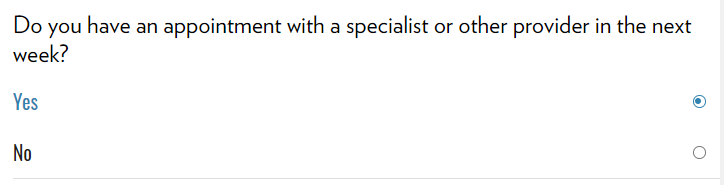


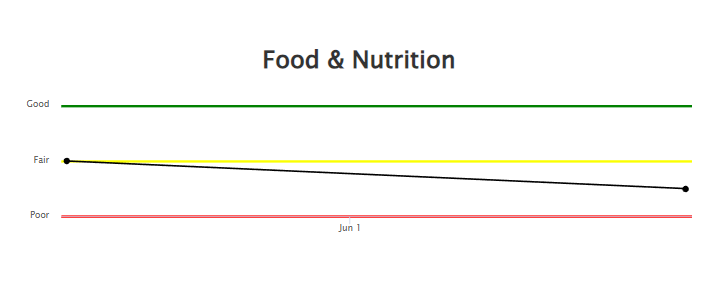


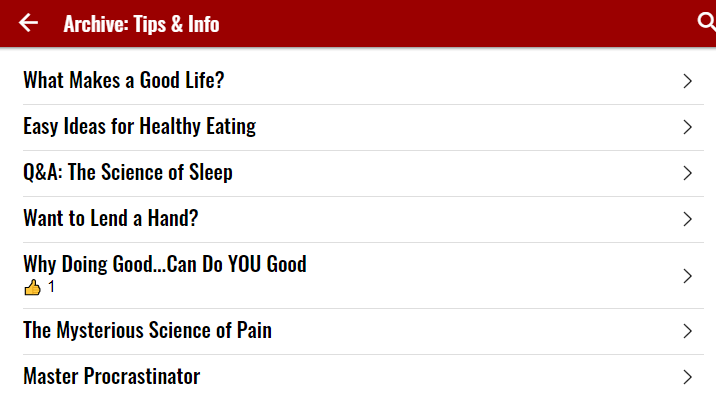

Supplement: Multimedia Appendix 2 [file aging_v7i1e56923_app2.docx]
